# Lupus-Prone Mice Fail to Raise Antigen-Specific T Cell Responses to Intracellular Infection

**DOI:** 10.1371/journal.pone.0111382

**Published:** 2014-10-31

**Authors:** Linda A. Lieberman, George C. Tsokos

**Affiliations:** Division of Rheumatology, Department of Medicine, Beth Israel Deaconess Medical Center, Harvard Medical School, Boston, Massachusetts, United States of America; University of Colorado School of Medicine, United States of America

## Abstract

Systemic lupus erythematosus (SLE) is characterized by multiple cellular abnormalities culminating in the production of autoantibodies and immune complexes, resulting in tissue inflammation and organ damage. Besides active disease, the main cause of morbidity and mortality in SLE patients is infections, including those from opportunistic pathogens. To understand the failure of the immune system to fend off infections in systemic autoimmunity, we infected the lupus-prone murine strains B6.*lpr* and BXSB with the intracellular parasite *Toxoplasma gondii* and survival was monitored. Furthermore, mice were sacrificed days post infection and parasite burden and cellular immune responses such as cytokine production and cell activation were assessed. Mice from both strains succumbed to infection acutely and we observed greater susceptibility to infection in older mice. Increased parasite burden and a defective antigen-specific IFN-gamma response were observed in the lupus-prone mice. Furthermore, T cell:dendritic cell co-cultures established the presence of an intrinsic T cell defect responsible for the decreased antigen-specific response. An antigen-specific defect in IFN- gamma production prevents lupus-prone mice from clearing infection effectively. This study reveals the first cellular insight into the origin of increased susceptibility to infections in SLE disease and may guide therapeutic approaches.

## Introduction

Systemic lupus erythematosus (SLE) is a debilitating disease, primarily affecting women, and presents with manifestations in most organs [1]. Aside from active disease, infections represent the major cause of morbidity and mortality [2], [3]. In fact, it has been reported 20–55% of all deaths of SLE patients result from infections [3]. Notwithstanding the fact that immunosuppressive drugs, routinely used in the treatment of patients with SLE, contribute to the increased rates of infections, inherent defects in the innate and acquired immune responses play an important role in susceptibility. For example, although T cells provide excessive help to B cells to produce autoantibodies, they are unable to raise proper cytotoxic responses [4] and they produce decreased levels of IL-2 [5]. Furthermore, autoantibodies against various cell components likely contribute to the increased incidence of infection in these patients [6], [7]. The mechanism of increased susceptibility to infection by SLE patients has not been well examined and this paper begins to unravel this phenomenon.

In addition to common infections, patients with SLE suffer from infections with opportunistic pathogens such as *Listeria monocytogenes, Cryptococcus neoformans, Pneumocystis carinii* and *Toxoplasma gondii* [8]. *T. gondii* is an intracellular parasite that often results in asymptomatic infection in healthy individuals as the parasite develops strategies to coexist with host cells [9]. Immunity to *T. gondii* is driven by IFN-gamma production [10]. SLE patients have higher titers of antibodies against *T. gondii* as compared to healthy controls [11] and a diagnosis of toxoplasmosis can easily be missed as the symptoms are similar to that of lupus cerebritis [12]. As with other CNS infections in SLE patients, *T. gondii* presents difficult diagnostic and therapeutic decisions.

To better understand the proclivity of SLE patients to suffer infections, we infected lupus-prone mice with *T. gondii*. Because individual lupus-prone murine strains do not epitomize the full spectrum of human disease, we infected two genetically-diverse lupus-prone murine strains (B6.*lpr,* BXSB) with this parasite and observed survival as well as elements of the cellular immune response. We report here that lupus-prone mice succumbed during the acute stage of infection due to increased parasite burden, independent of elevated systemic IFN-gamma levels in the serum. We found that *T. gondii*-infected mice display a severely depressed antigen-specific T cell IFN-gamma response that likely accounts for the increased mortality. This information sheds new light on the origin of increased susceptibility to infections in systemic autoimmunity and suggests the need for new approaches to mitigate infection-related morbidity and mortality.

## Materials and Methods

### Mice

Two strains of lupus-prone mice and appropriate controls were purchased for these studies. Female C57BL/6 mice were age and sex matched with B6.*lpr* mice and the lupus-prone male BXSB mice were age matched with female BXSB littermates (Jackson Laboratories, Bar Harbor, ME). Mice were randomly assigned to control or experimental group. Three to six mice per group were used for each experiment and replicated multiple times as indicated in the figure legend. CBA (Jackson Laboratories, Bar Harbor, ME) and Swiss Webster (Taconic, Germantown, NY) mice were used for *in vivo* maintenance of parasites. Mice were group housed in the barrier facility in the Center for Life Science at Beth Israel Deaconess Medical Center with a twelve-hour light/dark cycle.

### Parasites

The Me49 strain of *T. gondii* was used for these studies (ATCC, Manassas, VA). Parasites were maintained and passaged *in vivo* in Swiss Webster or CBA mice. Parasites were also maintained *in vitro* in HS27 human foreskin fibroblast cells (ATCC, Manassas, VA). Parasites were prepared from mouse brain isolates and diluted in phosphate buffered saline for injections.

### Survival Curves

Lupus-prone mice and appropriate controls were infected intraperitoneally with the Me49 strain of *T. gondii*. Infected mice were monitored daily for signs of lethargy, ruffling, and abnormal ambulation. If mice displayed any one of these criteria, they were then monitored twice daily. Mice were sacrificed by gas cylinder CO_2_ when they became moribund and that day post infection was considered the survival endpoint.

### ELISA, serum collection, urine collection, antibody treatment

Splenocytes from infected animals were stimulated *in vitro* with 5 ng/ml of IL-12 or 20 ug/ml of STAg for 72 h and supernatants were assayed for IFN-gamma by ELISA (eBiosciences, San Diego, CA). IFN-gamma and other cytokines were measured from serum collected from the tail vein by a multiplex bead assay (Bio-Rad, Hercules, CA). Anti-dsDNA was measured by ELISA from murine serum monthly to assess autoimmune status (Alpha Diagnostic International, San Antonio, TX). Urine was collected overnight in metabolic cages once a month and proteinuria was measured to monitor lupus-disease progression. Anti-PD-L1 (gift from Gordon Freeman Dana Farber Research Institute, Boston, MA) was administered intraperitoneally at 200 mg starting at day −1 before infection and every third day thereafter.

### Flow cytometry

Splenocytes or peritoneal exudate cells were surface stained with the following markers (CD3, CD4, CD8, NK1.1, B220, CD5, CD11-b, GR-1, CD11c, F4/80, IA/IE, H-2k, CD44, CD62L, CD80, CD86, PD-1, PD-L1, Tim-3, Ly6G, CD21, CD23, CD38, CD25, Fas, FoxP3; Biolegend, San Diego, CA) and cells were collected on an LSRII flow cytometer (Becton Dickinson, CA). Data was analyzed using FlowJo software (Treestar, Ashland, OR).

### Recall response

Spleens were removed from mice following sacrifice 7 days post-infection. Splenocytes were isolated and plated at 4 × 10^5^ cells/well in 96 well plates. Cells were stimulated with the parasite antigen STAg and/or IL-12 for 72 hours. STAg was prepared as previously described [13]. IFN-gamma production was measured by ELISA.

### Non-specific TCR stimulation

CD3^+^ T cells were purified from spleens of naïve B6.*lpr* and BXSB mice by negative selection (Pan T cell isolation kit II, Miltenyi, Auburn, CA) and stimulated with anti-CD3 (0.5ug/ml; eBiosceinces) and anti-CD28 (1 ug/ml; Biolegend) for 3 days. Supernatants were assayed for IFN-gamma production by ELISA.

### Parasite Burden

Cells were collected by peritoneal lavage with 5 ml cold PBS following the sacrifice of the mice and cytospins were prepared to determine parasite burden. Slides were stained with Hema3 Stain (Biochemical Sciences, Swedesboro, NJ) and sealed with mounting medium (Richard Allan Scientific, Kalamazoo, MI). Parasite burden was assessed by counting a minimum of 500 cells per cytospin in a blinded manner.

### T cell:DC mixing experiment

Lupus-prone and control mice were infected with *T. gondii* and 7 days later spleens were removed and splenocytes were prepared. CD11c^+^ cells were isolated by magnetic separation using positive selection (CD11c^+^ isolation kit, Miltenyi, Auburn, CA). The flow-through was collected and CD3^+^ cells were isolated by negative selection from the same animal (Pan T cell isolation kit II, Miltenyi, Auburn, CA). Cells were plated in 96 well plates at a ratio of 5 T cells to 1 dendritic cell (T-3×10^5^: DC-6×10^4^). Antigen was added to the wells (STAg 20 ug/ml) and the plate was incubated for 72 hours at 37°C, 5% CO_2_. Supernatants were assayed for IFN-gamma production by ELISA (eBioscience, San Diego, CA).

### Statistical analysis

Unpaired two-tailed Student *t* tests were calculated using PRISM software (GraphPad). A *p* value of <0.05 was considered significant.

### Ethics Statement

All murine work was reviewed and approved by the IACUC at Beth Israel Deaconess Medical Center. Beth Israel Deaconess Medical Center is AAALAC accredited and complies with all federal, state, and local laws. These studies were carried out under IACUC protocols 101–2009 and 069–2012.

## Results

### Lupus-prone mice are susceptible to infection with *T. gondii*

We used a model of *T. gondii* infection to investigate whether inherent immune dysfunction in lupus-prone mice leads to increased susceptibility to infection. Because none of the commonly used lupus-prone murine strains perfectly mimic human SLE disease, we decided to study two different mouse strains. The mice we chose to investigate were B6.*lpr* (mutated lymphoproliferation (*lpr*) gene), and the BXSB strain (male mice have the Y chromosome linked autoimmune accelerator gene (*Yaa*)). The B6.*lpr* mice are defective in Fas-mediated signaling [14], while the BXSB mice overexpress *Tlr7* [15]. The onset of lupus disease in these mice occurs at different time points, therefore we infected mice 1–3 months before the onset of the disease to ensure that the results would not be complicated by immune abnormalities and organ damage imposed by disease activity. Accordingly, the mice studied had increased levels of anti-dsDNA antibodies but they had not yet developed proteinuria.

**Figure 1:**
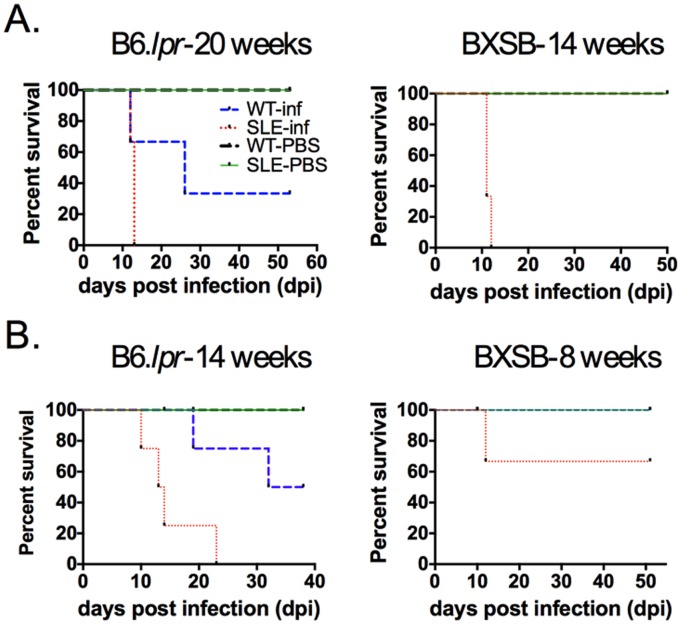
Lupus-prone mice succumb to acute infection with *T. gondii*. (A) Two strains of SLE-prone mice exhibiting exacerbated autoimmune markers but no lupus pathology were infected with 20 Me49 i.p. and survival was monitored. B6.*lpr* mice were age and sex matched with C57BL/6 mice. Male BXSB mice were age-matched with females of the colony who do not develop lupus-like disease. All lupus-prone mice succumbed to infection within the first 12 days of infection. (B) Younger mice were infected for the above strains and it was observed that they had increased survival. Each survival curve represents at least two replicates of 3–6 mice per group.

First we infected B6.*lpr* mice intraperitoneally with *T. gondii* at 20 weeks of age as they displayed a significant increase in circulating anti-dsDNA antibodies as compared to wild type controls, but they did not exhibit any proteinuria. Similarly, BXSB mice were infected at 14 weeks of age. We found that although they are genetically different, both SLE-prone mice rapidly succumbed to acute infection within twelve days post-infection (Figure 1A). This differs from previously published data in which NZBWF1 mice were orally infected with *T. gondii*, resulting in a chronic infection [16]. This variance may have to do with the different genetic background of those mice and/or the route of infection as peroral infection often results in chronic disease.

Additionally, we infected younger mice to determine whether susceptibility to infection is affected by age as mice develop more autoimmune manifestations with age. B6.*lpr* mice were infected at 14 weeks of age and BXSB mice were infected at 8 weeks of age. We found that mice expressing lower levels of anti-dsDNA (therefore less autoimmunity) were less susceptible to infection compared to older mice suggesting immunosuppression increases with disease progression (Figure 1B). All of the following studies were conducted on B6.*lpr* mice at 20 weeks of age and BXSB mice at 14 weeks of age.

### Lupus-prone mice display an increased parasite burden following infection

**Figure 2:**
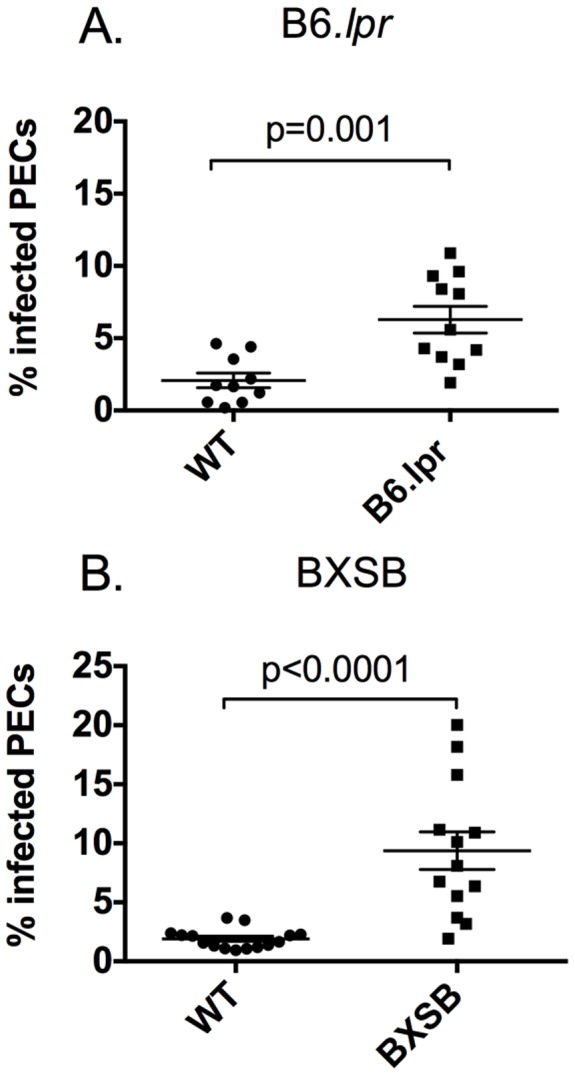
Parasite burden is increased in lupus-prone mice. Mice were sacrificed 7–10 days following infection and peritoneal lavage was performed. A cytospin was prepared from each mouse and then the percent of infected cells was quantified. (A) B6.*lpr* mice showed a significant increase in parasite burden 9 dpi. (B) BXSB mice had significantly increased parasite burden 7 dpi. Each experiment was carried out 2–3 times with 3–6 mice per group.

Previous studies have established that many immunocompromised mice succumb to acute *T. gondii* infection when they are unable to control parasite replication, an event linked to defects in cytokine production or function. We measured parasite burden in the peritoneum of infected mice and in B6.*lpr* we found a significantly higher parasite burden in mice 9 days post infection (p = 0.001) (Figure 2A). An increased parasite burden was observed 7 days post infection in the BXSB mice (p<0.0001) (Figure 2B). These data indicate that lupus-prone mice are unable to effectively control parasite replication.

### Systemic IFN-gamma does not sufficiently protect against *T. gondii* infection

**Figure 3:**
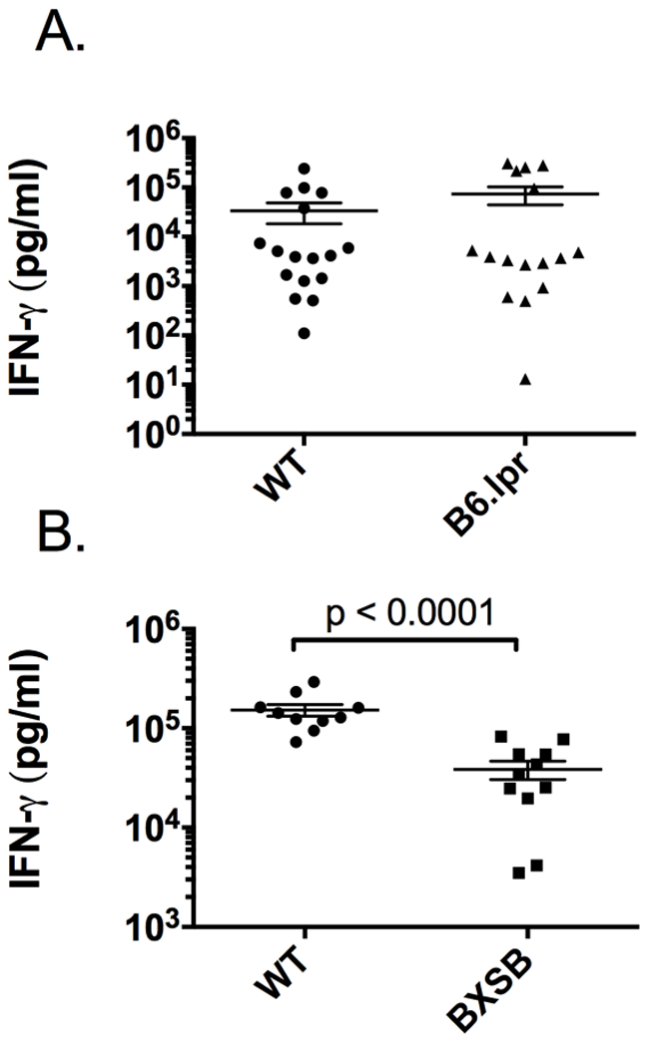
Systemic IFN-gamma does not sufficiently protect lupus-prone mice. IFN-gamma levels were measured in the serum of lupus-prone mice 7 days post infection (dpi) by multiplex. No significant differences were found between infected B6.*lpr* mice and infected WT controls (A). BXSB mice had reduced levels of systemic IFN-gamma (B). B6.*lpr* mice were 20 weeks of age and BXSB mice were 14 weeks of age. Each experiment was carried out at least 3 times with 3–6 mice per group.

The major route of resistance to *T. gondii* infection is IL-12 driven IFN-gamma production. Increased parasite burden is often associated with decreased IFN-gamma production [17]. We examined serum cytokine levels at the peak of IFN-gamma production, 7 days after infection, and found no significant difference in the systemic levels of IFN-gamma in B6.*lpr* mice (Figure 3A). In fact, the levels of IFN-gamma in the B6.*lpr* mice after infection tended to be higher than that of wild type mice, though this difference did not reach significance. Conversely, IFN-gamma levels in BXSB mice were significantly decreased (Figure 3B). This suggests IL-12 levels may also be decreased, but we found no defect in systemic IL-12 (data not shown).

### T cell antigen specific IFN-gamma production is decreased in lupus-prone mice following *T. gondii* infection

**Figure 4:**
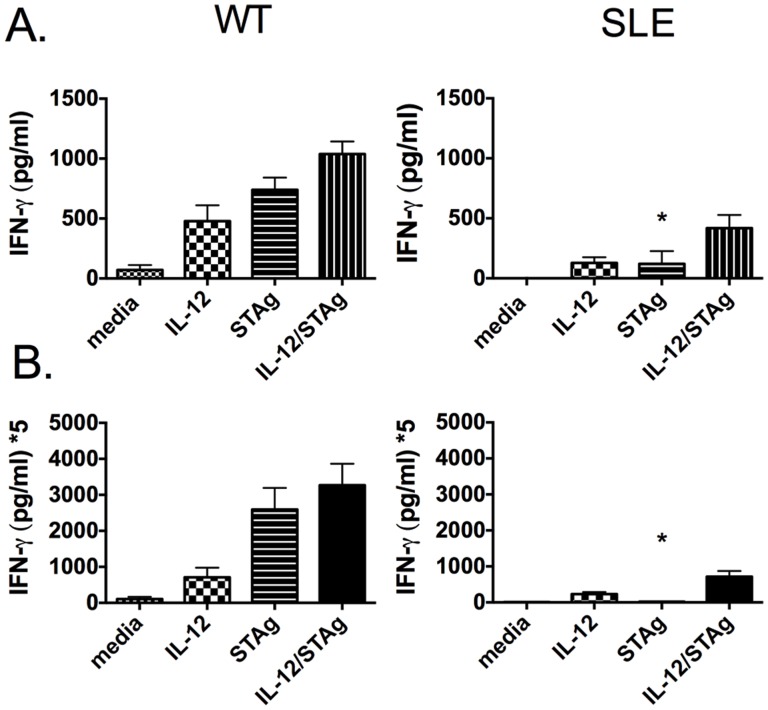
Antigen-specific response is defective in lupus-prone mice. Splenocytes were collected from infected mice 7 dpi and were stimulated 3 days in vitro with either IL-12 (5ng/ml), STAg (20 ug/ml), or IL-12+ STAg. Supernatants were assayed by ELISA for IFN-gamma production. A significant decrease in antigen-specific IFN-gamma was observed (*). (A) B6.*lpr* (B) BXSB. B6.*lpr* mice were 20 weeks of age and BXSB mice were 14 weeks of age. This experiment is representative of 2–3 individual experiments with 3–4 mice per group.

We examined activation markers on various cell types from the spleen and the peritoneum of infected (and uninfected) lupus-prone mice but found no significant differences in distribution or activation markers as compared to wild type mice (data not shown). Since T cell activation appeared normal as evidenced by no significant differences in the percentage of CD3^+^CD4^+^ CD44^hi^CD62L^lo^ or CD3^+^CD8^+^CD44^hi^CD62L^lo^ cells, we asked whether T cells from lupus-prone mice were functionally competent. To address this question, we assessed the ability of these cells to respond to specific antigen stimulation. We stimulated splenocytes from infected mice with *T. gondii*-specific antigen (STAg) for 3 days *in vitro* and measured IFN-gamma production in the culture supernatants. We found that cells from infected B6.*lpr* (Figure 4A) and BXSB mice (Figure 4B) produced greatly reduced levels of antigen-specific IFN-gamma indicating a defective antigen recall response.

**Figure 5:**
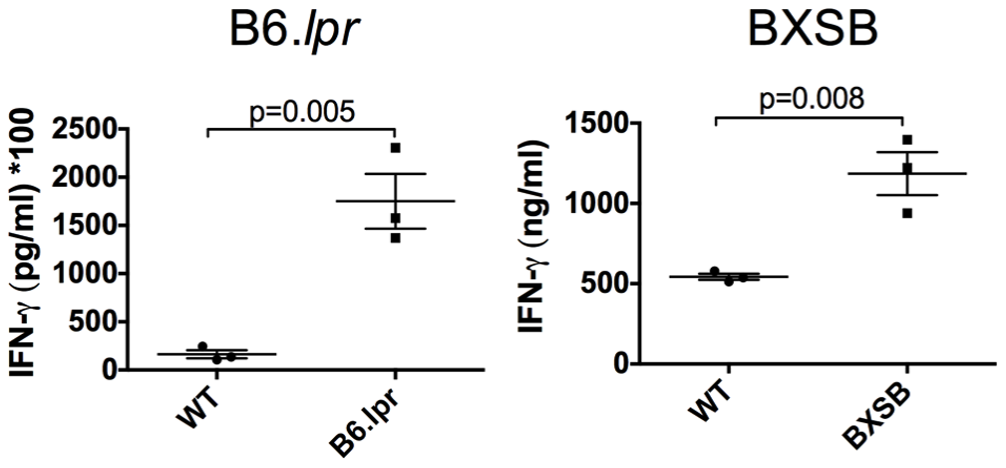
TCR-stimulation is not defective in lupus-prone mice. T cells were isolated from splenocytes of either B6.*lpr* or BXSB mice and the appropriate controls. Cells were stimulated with anti-CD3/anti-CD28 for 72 hours and supernatants were assayed for IFN-gamma production by ELISA. The IFN-gamma response to non-specific TCR stimulation was intact in these mice. B6.*lpr* mice were 20 weeks of age and BXSB mice were 14 weeks of age. This experiment is representative of two individual experiments.

To ensure that this response was indeed antigen specific and not due to a non-specific T cell defect in these mice, CD3^+^ T cells were purified from spleens of naïve B6.*lpr* and BXSB mice and stimulated with anti-CD3 and anti-CD28 for 3 days. Supernatants were assayed for IFN-gamma production and we did not find any defect in the ability of these cells to respond to non-specific TCR stimuli (Figure 5). In fact, in both B6.*lpr* and BXSB mice, we observed an increased IFN-gamma response as compared to the wild type controls. Similarly, an increased IFN-gamma response has been reported following TCR stimulation of peripheral T cells from SLE patients [18].

### An intrinsic T cell defect leads to decreased antigen-specific response

**Figure 6:**
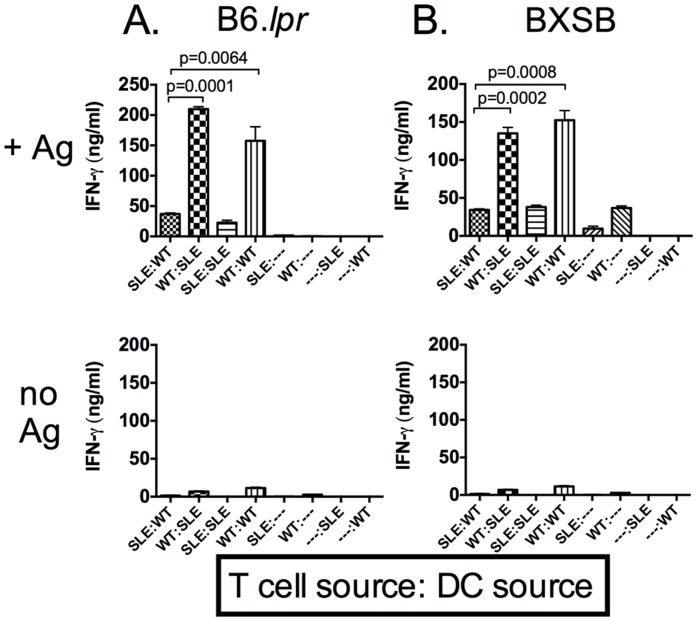
An intrinsic T cell defect is responsible for the decrease in antigen-specific IFN-gamma production. CD3^+^ T cells and CD11c^+^ DCs were isolated from splenocytes of infected mice 7 dpi. Cells were mixed at a ratio of 5∶1 (T:DC) and incubated for 72 hours +/− STAg. IFN-gamma was measured from supernatants by ELISA. (A) B6.*lpr*; (B) BXSB; each assay was performed 3 times in triplicate. B6.*lpr* mice were 20 weeks of age and BXSB mice were 14 weeks of age.

The decreased antigen-specific response observed in lupus-prone mice is due to decreased IFN-gamma production by T cells. To investigate whether this is due to an intrinsic T cell defect or the result of defective antigen presentation, we purified T cells and dendritic cells (DCs) from spleens of infected wild type or lupus-prone mice and mixed them *in vitro* in the presence or absence of *T. gondii* antigen. There was a baseline level of IFN-gamma produced in the absence of STAg (Figure 6 lower portion) and this is to be expected since the parasite is present in the splenocyte cultures isolated from infected animals. We found that T cells from lupus-prone mice mixed with DCs from wild type mice produced less IFN-gamma as compared to wild type T cells mixed with DCs from lupus-prone mice which produced similar levels of IFN-gamma as wild type T cells mixed with wild type DCs (Figure 6 upper portion). Therefore, we conclude that antigen presentation is intact in the lupus-prone mice, but these mice harbor an intrinsic T cell defect leading to decreased IFN-gamma production.

## Discussion

One of the leading causes of morbidity and mortality in SLE patients is infection. It is well known that the immune system of SLE patients is dysregulated but there has been little insight as to how it responds to challenge with infectious organisms. The use of steroids and other immunosuppressive drugs in SLE patients complicates the ability to separate the effects of the drugs from the natural dysregulation of the immune response triggered by disease. Lupus-prone mice allow us to parse the immune response to infectious agents in the context of autoimmunity. We show in this study that lupus-prone mice display increased susceptibility to infection which is accentuated as disease progresses. To our surprise, lupus-prone mice succumbed acutely to infection with the prototypic Th1-inducing pathogen *T. gondii*; this occurred in both B6.*lpr* mice and BXSB mice. We observed decreased systemic IFN-gamma production in the BXSB mice therefore it was not surprising that there was a decreased ability to control parasite burden as it has been well established that IFN-gamma is necessary for control of this parasite. Furthermore, it has previously been reported that BXSB mice are susceptible to acute infection with the intracellular parasite *Trypanosoma cruzi* [19]. Conversely, the B6.*lpr* mice did not have decreased systemic IFN-gamma, therefore the dichotomy between the increased levels of IFN-gamma in the serum of B6.*lpr* mice following infection (similar to what is seen in wild type mice), and the inability to contain parasite replication, was unexpected. This may be explained by considering the sources of IFN-gamma during *T. gondii* infection. During early infection, systemic IFN-gamma comes from multiple cellular sources with NK cells being a major producer of IFN-gamma. While this IFN-gamma will be protective, it does not provide sufficient protection for the mice to survive beyond acute infection (approximately two weeks in wild type mice) [20]. Antigen-specific IFN-gamma production from T cells is necessary for protection against this pathogen [21].

Interestingly, patients with SLE are susceptible to infection with other Th1 pathogens such as *Salmonella spp.* [6] though they have elevated levels of IFN-gamma [22], [23]. This may be explained by a decreased ability of IFN-gamma to bind the IFN-gamma receptor or perhaps a defect in signaling through this receptor. It has been reported by one group that polymorphisms in the IFN-gammaR may increase the likelihood of SLE development and it has been shown that these polymorphisms can lead to an alteration in receptor function, resulting in a decreased response to IFN-gamma [24], [25]. Conversely, another group concluded these polymorphisms are not associated with SLE in a different patient population [26].

The significantly decreased production of antigen-specific IFN-gamma in response to infection was unexpected. The expression of autoimmunity has been found to be dependent on the presence of IFN-gamma particularly during late stages of disease. Elevated IFN-gamma levels contribute to tissue damage and it has been reported that lupus-prone mice in which IFN-gamma or IFN-gammaR has been deleted display reduced disease and mortality [27]. From our studies, it appears that non-specific IFN-gamma production found in lupus-prone mice is not sufficient to provide defense against infectious agents; survival may depend on antigen-specific IFN-gamma. As shown in Figure 1B, mice that have not yet developed significant autoimmunity survive longer than mice that have established autoimmune disease. We questioned if this increased protection was reliant on antigen-specific IFN-gamma production. Interestingly, recall responses from splenocytes of infected 14-week old B6.*lpr* mice or 8-week old BXSB mice displayed no defect in antigen specific IFN-gamma production (data not shown) suggesting this T cell function is integral to a successful immune response to *T. gondii*.

Upon observation that IFN-gamma was decreased in recall cultures, we wanted to know if this was due to a defect in antigen presentation or if it was a result of T cell dysfunction. We found that T cells are directly responsible for the defect in antigen specific IFN-gamma production as T:DC co-cultures revealed that antigen presentation is intact in these lupus-prone mice. Studies from our group and others have identified various defects in T cell signaling in SLE T cells and this may contribute in part to the observations made in these studies [28]. It should be considered that though the same number of T cells were added to the mixing cultures from either wild type or lupus-prone mice, the ratio of parasite-specific cells to other T cells present may be different between mouse strains. The lupus-prone mice may not increase their number of *T. gondii*-specific T cells as robustly as wild type mice. New tools may allow us to identify the percentage of antigen-specific T cell clones in the future.

During this study we found that lupus-prone mice trended towards increased expression of PD-1 on CD4^+^ T cells from infected mice as compared to T cells from infected wild type mice (data not shown). We considered that T cells had become refractive to stimulation due to the increased levels of PD-1, a molecule known to be upregulated on functionally exhausted T cells [29]. Furthermore, it has been reported that the PD-1:PD-L1 pathway plays a role in inhibiting the functionality of CD8^+^ T cells during chronic *T. gondii* infection [30]. We indirectly blocked PD-1 signaling by injecting anti-PD-L1 into B6.*lpr* mice prior to and following infection with *T. gondii*. Though it has been reported that infecting PD-L1^−/−^ mice results in better outcomes for some infections, we found that blocking PD-L1 did not affect survival of lupus-prone mice. Similarly, it has been reported that blocking PD-L1 during acute *L. monocytogenes* infection does not enhance the immune response [31]. We concluded that the defect we observed in the lupus-prone mice was not likely due to functional exhaustion, though it should be noted that we did not block secondary ligands to PD-1, such as PD-L2 or PD-L3.

Our data present novel insight into the cellular immune events that account for increased susceptibility of lupus-prone mice to infection. At the translational level, our data suggest that in addition to antibiotics, the correction of failing T cell function in SLE patients should produce more favorable clinical outcomes following infections.

## Supporting Information

Table S1 **ARRIVE guidelines checklist.**
(PDF)
